# Management of Irreparable Posterosuperior Rotator Cuff Tears—A Current Concepts Review and Proposed Treatment Algorithm by the AGA Shoulder Committee

**DOI:** 10.3390/jpm13020191

**Published:** 2023-01-21

**Authors:** Jonas Pogorzelski, Marco-Christopher Rupp, Bastian Scheiderer, Lucca Lacheta, Benedikt Schliemann, Jakob Schanda, Philipp Heuberer, Marco Schneider, Michael Hackl, AGA Shoulder Committee—Rotator Cuff, Olaf Lorbach

**Affiliations:** 1 Department of Orthopedic Sports Medicine, Technical University of Munich, Klinikum rechts der Isar, Ismaninger Str. 22, 81675 Munich, Germany; 2 Private Practice OC Erlangen-Ebermannstadt, Nägelsbach Str. 25b, 91052 Erlangen, Germany; 3 Herz Jesu Hospital Münster-Hiltrup, Westfalen Str. 109, 48165 Münster, Germany; 4 Private Practice Dr. Schanda, Rochusgasse 17/13, 1030 Vienna, Austria; 5 Private Practice OrthoCare, Kurbad Str. 14, 1100 Vienna, Austria; 6 University of Witten/Herdecke, Alfred-Herrhausen-Straße 45, 58455 Witten, Germany; 7 Department of Trauma-, Hand- and Elbow Surgery, University Hospital of Cologne, Kerpener Str. 62, 50937 Cologne, Germany; 8 Schoen-Clinic Lorsch, Department of Shoulder Surgery and Sports Traumatology, Wilhelm-Leuschner-Straße 10, 64653 Lorsch, Germany

**Keywords:** irreparable, rotator cuff tear, joint preservation, partial repair, superior capsular reconstruction, tendon transfer, balloon system, treatment algorithm, guideline, expert consensus

## Abstract

Posterosuperior rotator cuff tears range among the most common causes of shoulder complaints. While non-operative treatment is typically reserved for the elderly patient with low functional demands, surgical treatment is considered the gold standard for active patients. More precisely, an anatomic rotator cuff repair (RCR) is considered the most desirable treatment option and should be generally attempted during surgery. If an anatomic RCR is impossible, the adequate choice of treatment for irreparable rotator cuff tears remains a matter of debate among shoulder surgeons. Following a critical review of the contemporary literature, the authors suggest the following evidence- and experience-based treatment recommendation. In the non-functional, osteoarthritic shoulder, treatment strategies in the management of irreparable posterosuperior RCT include debridement-based procedures and reverse total shoulder arthroplasty as the treatment of choice. Joint-preserving procedures aimed at restoring glenohumeral biomechanics and function should be reserved for the non-osteoarthritic shoulder. Prior to these procedures, however, patients should be counseled about deteriorating results over time. Recent innovations such as the superior capsule reconstruction and the implantation of a subacromial spacer show promising short-term results, yet future studies with long-term follow-up are required to derive stronger recommendations.

## 1. Introduction

In the management of tears of the posterosuperior rotator cuff, characterized by tears of the tendons of the supraspinatus and infraspinatus muscle, there exists consensus that an anatomic rotator cuff repair (RCR) is the most desirable treatment option in active patients in the absence of severe degenerative changes [1]. However, the optimal management of irreparable posterosuperior rotator cuff tears (RCTs) still remains a topic of debate among shoulder surgeons. While the definition may vary, a posterosuperior RCT is typically considered “irreparable” if a primary repair of the affected tendon to its anatomic footprint is impossible despite sufficient surgical release and the mobilization of the tendon. More specifically, the widely accepted definition by Gerber et al. [2] defines a RCT to be “irreparable” if it is impossible to achieve anatomic fixation of the torn posterosuperior tendons in <60° of glenohumeral abduction despite adequate tendon release. Factors that increase the risk of a posterosuperior RCT to be “irreparable” include decreased tendon elasticity [3], fatty infiltration [4], anterior-posterior tear size [5] and a progression to cuff arthropathy [6] that results in alterations of the glenohumeral biomechanics [7]. The term “massive” RCT—describing full thickness tears of ≥2 tendons, retraction to glenoid and/or exposure of 67% of the greater tuberosity [8]—is often incorrectly used and interchanged with the term “irreparable” and should be used separately.

If an RCR is deemed impossible prior to surgery due to an irreparable situation, treatment options such as non-operative management, the debridement of the rotator cuff with or without the tenotomy or tenodesis of the long head of the biceps tendon (LHBT), partial repair, tendon transfers (TTs), the implantation of a subacromial biodegradable spacer (SBS), superior capsular reconstruction (SCR) and the implantation of a reverse total shoulder arthroplasty (RTSA) are considered viable alternatives [2,9,10,11,12,13,14]. Specific advantages and disadvantages have been reported for each of these procedures. Consequently, the surgeon has to determine the ideal treatment approach based on patient-related, anatomic, and injury-related predictors according to an individualized treatment algorithm.

The purpose of this article was to review the current literature and to provide a current concepts overview of available contemporary treatment options for irreparable posterosuperior RCTs as well as to present an evidence- and experience-based clinically applicable treatment algorithm.

## 2. Non-Operative Treatment

Given a failure rate of up to 70% after RCR of chronic and massive tears [15] and acknowledging that pain relief and functional improvement do not necessarily correlate with successful structural healing of the tendon [16], non-operative treatment has been proposed as the treatment of choice for atraumatic RCT [17]. In the setting of irreparable RCTs, a special physiotherapy concept termed “anterior deltoid re-education” (ADR) has been proposed, which consists of an exercise regimen to rehabilitate the deltoid muscle to compensate for the deficient rotator cuff. This concept has been shown to result in acceptable outcomes in a group of comorbid, elderly patients in combination with subacromial injections of local anesthetics and steroids as well as an oral therapy with non-steroidal anti-inflammatory drugs [18]. More specifically, in a collective of 17 patients (mean age: 80 years), there was a statistically significant improvement in the mean Constant Score, a score combining patient subjective criteria as well as objective functional assessments [19], from 26 points prior to treatment (range, 8–41 points) to 60 points (range, 43–77 points) at a mean follow-up of nine months (*p* < 0.05). The range of motion in forward elevation improved from a mean of 40° (range 30–60°) prior to treatment to a mean of 160° (range 150–180°) [18]. However, a recent study investigating patient-reported outcomes after the same concept of ADR in 30 patients with a mean age of 74 years (range 55–89 years) has shown that only 12 patients improved by more than 20 points in the American Shoulder and Elbow Surgeons (ASES) Score, the threshold that is considered a successful non-operative treatment [20]. Persisting pain and kinematic dysfunction of the glenohumeral joint were the most common reasons for failure. In summary, careful patient selection is necessary when non-operative treatment for irreparable RCT is administered, as patients should be willing to accept discomfort and functional deficits of their shoulder in exchange for avoiding the risks of surgery. Non-operative treatment in the management of irreparable RCT is considered to yield the best outcomes in elderly patients with low functional demands or in patients for whom surgical treatment is contraindicated (Table 1).

## 3. Arthroscopic Debridement, Tenotomy/Tenodesis of the Long Head of the Biceps Tendon and (Reversed) Subacromial Decompression

Irreparable RCT may also be treated by an arthroscopic debridement of the remaining rotator cuff in combination with a (reversed) subacromial decompression (SAD) and a tenotomy or tenodesis of the LHBT (Table 2) [21,22,23]. While lesions of the LHBT have been identified as a source of persistent pain that can be resolved with LHBT tenotomy, the purpose of arthroscopic SAD is to create a smooth, non-impinging acromiohumeral articulation by creating space, removing osteophytes and bony irregularities. A “reverse SAD”, also known as tuberoplasty, has been introduced in order to protect the coracoacromial ligament—a structure at risk in regular SAD—to prevent an antero-superior escape of the humeral head, while equally ensuring a smooth acromiohumeral articulation [24,25]. Debridement combined with (reverse) SAD has been shown to result in decreased pain levels while maintaining residual rotator cuff strength (i.e., no improvement in shoulder strength). Low physical demand, sufficient residual shoulder function, older patient age and pain as the chief complaint are the main indications for arthroscopic debridement combined with (reverse) SAD and LHBT tenotomy/tenodesis. As such, a contemporary study in 19 patients (mean age 68 years) with symptomatic massive RCT reports significant improvements in functional outcomes, with an improvement in ASES Score of more than 30 points and a significant pain relief after arthroscopic debridement with SAD and the tenotomy of the LHBT after a minimum 10-year follow-up [23]. However, in this cohort 26% of patients failed and underwent RTSA during follow-up, emphasizing the importance of careful patient selection and preoperative counseling [23].

## 4. Partial Rotator Cuff Repair

Even if a complete anatomical repair is impossible, a partial repair of an irreparable RCT is an option to restore the rotator cuff’s force couple, which is created by the subscapularis muscle anteriorly and the infraspinatus and teres minor muscles posteriorly. Even in cases of a torn supraspinatus tendon, a sufficient concavity compression of the humeral head into the glenoid can be obtained by a balanced force couple and thus prevent the superior migration of the humeral head [26]. Several authors have published their short- and mid-term clinical outcomes following partial repairs of massive RCTs. Shon et al. [27] reported patient-reported outcomes after partial repairs of irreparable posterosuperior RCTs combined with or without subscapularis tendon repair in 31 patients preoperatively and at 1 and 2 years after surgery. Despite significant improvements from preoperatively to postoperatively, a slight deterioration was found from 1 to 2 years postoperatively (ASES 76 vs. 74, SST 6.6 vs. 6.1, VAS 2.1 vs. 3.2). In addition, even when considering the initial improvement, subjective patient-reported satisfaction was reported as “rather the same” or “dissatisfied” in 15 patients (48%). Teres minor fatty infiltration was identified as an independent factor affecting patient-reported satisfaction. Slightly better mid-term results were reported by Cuff et al. in 28 patients who underwent partial repairs and LHBT tenotomy at a minimum 5-year follow-up with a satisfaction rate of 75% [28]. Despite significant improvements in ASES Score (47 to 79), SST (5.7 to 9.1) and VAS for pain (6.9 to 1.9) at final follow-up compared to preoperatively, the failure rate was as high as 29%. Of note, failure was defined as an ASES score of <70 points, loss of active elevation over 90° or revision arthroplasty surgery. Summarizing these findings, initial favorable clinical results can be expected in patients whose primary complaints are pain and weakness in the setting of good preoperative shoulder function without prior osteoarthritic changes. However, patients should be counseled that clinical outcomes may deteriorate over time (Table 3).

## 5. Tendon Transfers

Tendon transfers such as the latissimus dorsi tendon transfer (LDTT) or the lower trapezius tendon transfer (LTTT) are considered viable treatment options in young patients without glenohumeral osteoarthritis (OA) but with functional deficits, especially in terms of external rotation, caused by an irreparable posterosuperior RCT (Table 4). As the coronal force couple is disrupted in this situation, higher forces are required by both the deltoid and the intact muscle-tendon units of the remaining rotator cuff to stabilize the humeral head. As these tears progress, this results in a superior migration of the humeral head and shoulder dysfunction, in particular a lack of external rotation [29].

### 5.1. Latissimus Dorsi Tendon Transfer

In its physiologic function, the latissimus dorsi muscle-tendon unit acts to adduct, internally rotate and extend the humerus. Its large muscle excursion makes the latissimus dorsi feasible for a muscle transfer procedure [30]. Transferring the muscle-tendon unit from its native anterior insertion at the mid-bicipital groove to posterosuperiorly to the greater tuberosity allows the tendon to close the rotator cuff defect. Furthermore, in this setting, the latissimus acts as an external rotator and a depressor of the humeral head [31], which restores the coronal force couple by taking over the biomechanical function of the posterosuperior rotator cuff tendons and improves glenohumeral function. However, the postoperative results reported after this procedure are variable [30,32,33,34,35,36,37,38,39,40,41,42]. Most studies evaluating clinical outcomes after LDTTs have reported a significant reduction in pain and an improvement in shoulder function. A systematic review by Namdari et al. [43] analyzed 10 studies between 1992 and 2010 to determine the expected outcomes, outcome predictors and complications of LDTTs. Patients had a frequency-weighted mean adjusted Constant Score of 45.9 preoperatively compared with 73.2 postoperatively (*p* < 0.001). Additionally, the frequency-weighted mean active forward elevation improved from 101.9° preoperatively to 137.4° postoperatively (*p* < 0.001), while the external rotation improved from 16.8° to 26.7° (*p* < 0.001). The overall reported complication rate was 9.5%, including surgical site infection, neurapraxia, hematomas, wound dehiscence and tears of the transferred tendon. Predictors of favorable outcomes included a lower degree of teres minor fatty infiltration, an LDTT as a primary procedure and the presence of an intact subscapularis tendon [43].

### 5.2. Combined Latissimus Dorsi and Teres Major Tendon Transfer (L’Episcopo Technique)

In 1934, L’Episcopo introduced the combined latissimus dorsi and teres major tendon transfer to regain external rotation in pediatric patients with plexus paralysis. Comparable to isolated LDTT, the transfer of both tendons allows the closing of the rotator cuff defect and allows them act as an external rotator and a humeral head depressor. Especially in patients where the teres minor muscle is already degenerated, the combined teres major and latissimus dorsi tendon transfer might be beneficial to balance the rotator cuff’s compromised force couple [33]. Lichtenberg et al. [44] compared clinical outcomes between isolated LDTT and combined teres major and latissimus dorsi tendon transfer at a mean follow-up of 6 years. Significant improvements in Constant Score and active range of motion were found in 17 patients in each group. However, group comparison showed significantly better active flexion and abduction for patients undergoing isolated LDTT compared to patients undergoing combined teres major and latissimus dorsi tendon transfer. In addition, there was no progression in the degree of rotator cuff arthropathy in patients who underwent isolated LDTT [44]. Combined, these findings suggest it is preferable to rely on the isolated LDTT technique rather than a combined LDTT and teres major tendon transfer as the default technique.

### 5.3. Lower Trapezius Tendon Transfer (LTTT)

The theoretical advantage of the lower trapezius tendon transfer (LTTT) compared to the LDTT is the synergistic function of the lower trapezius muscle and the infraspinatus muscle, sharing a similar force vector trajectory. Accordingly, both muscles contribute to scapular retraction and glenohumeral external rotation [45]. Compared to the LDTT, the LTTT is considered to be more anatomic, which reduces the need for intensive retraining during the postoperative recovery period. However, as the lower trapezius tendon has a short excursion, an additional tendon graft such as the semitendinosus tendon is required to cover the distance between its native insertion site at the scapular spine and the footprint of the torn rotator cuff at the greater tuberosity [46]. Recently, three studies assessed clinical outcomes of an LTTT as treatment for irreparable posterosuperior RCT [46,47,48]. In 2016, Elhassan et al. [48] reported the outcomes of 33 patients with an average age of 53 years following LTTT prolonged by Achilles tendon allograft. At an average follow-up of 47 months, 32 patients showed significant improvements in the Subjective Shoulder Value (SSV; 54% preoperatively, 78% postoperatively; *p* < 0.01) and Disabilities of Arm, Shoulder and Hand (DASH) Score (52 ± 19 preoperatively, 18 ± 10 postoperatively; *p* < 0.01). One patient failed and required shoulder fusion subsequently. All successfully treated patients improved their ability for glenohumeral external rotation. Interestingly, patients with more than 60° of preoperative flexion had more improvement in range of motion than patients with less than 60° of glenohumeral flexion [48]. A more recent study showed similar results in 41 patients undergoing arthroscopically assisted LTTT augmented with an Achilles tendon allograft [47]. At a mean follow-up of 14 months, about 90% of the patients reported significant improvement in all outcome measures, including pain, shoulder motion and patient reported outcomes. Of note, the authors reported eight early complications with four of them being peripheral nerve symptoms due to immobilization in a custom external rotation brace for a total of 6–8 weeks postoperatively. All symptoms resolved spontaneously over a 1–3-month period after removing the brace [47]. Valenti and Werthel [46] investigated 14 patients with a mean age of 62 years undergoing LTTT with semitendinosus tendon augmentation to reconstruct irreparable posterosuperior RCTs. At a minimum 18-month follow-up, all clinical scores improved significantly with the mean Constant Score improving from 35 ± 15 to 60 ± 9 points, mean VAS for pain decreasing from 7 to 2 points and mean SSV improving from 40% to 70%. Two of the 14 patients suffered from minor early complications (hematoma and surgical site infection) requiring revision surgery [46]. Compared to the reconstruction of passive stability by the means of an SCR, the LTTT was superior in terms of functional improvement (ASES score of 84.8 ± 7.6 vs. 76.8 ± 20.3, respectively; *p* = 0.045), patient satisfaction, progression of arthritis (SCR: 22.7% vs. with LTTT: 2.8%) and graft integrity (retear rate: SCR 63.6% vs. LTTT: 8.3%) at minimum 2 years follow-up [49].

## 6. Superior Capsular Reconstruction (SCR)

The SCR has recently been introduced as an alternative treatment for irreparable posterosuperior RCT that is less invasive compared to tendon transfer surgery (Table 5). Biomechanically, the superior capsule of the glenohumeral joint contributes to static stabilization and is commonly disrupted when the supraspinatus and infraspinatus tendons are injured [50]. The technique of SCR in its current form was developed in Japan by Mihata et al. [51], who used a fascia lata autograft to reconstruct the superior capsule as treatment for an irreparable RCT. Today, a variety of allografts, including human acellular dermal allografts, are available [52,53]. In general, the graft prevents the superior migration of the humeral head, thereby maintaining the glenohumeral fulcrum if the force couple is still intact. Commonly accepted contraindications include high-grade glenohumeral OA (Hamada > 2), deltoid weakness, and irreparable subscapularis tears [54,55]. The main indications for an SCR include pseudoparesis (defined as active scapular plane abduction of more than 45° and less than 90°), an intact subscapularis tendon or reparable subscapularis tendon tear, and the absence of an external rotation lag sign [56,57].

Two recent systematic reviews of early postoperative clinical outcomes reported convincing clinical results in an early stage [55,58]. Clinical scores including the ASES Score improved significantly across all studies with mean postoperative scores ranging from 70–90 points [55,58]. Failure, mainly defined as graft rupture, occurred in 13–14% of cases [55,58]. This is in contrast to another recent systematic review and meta analysis that also reported satisfactory clinical and radiological results at a minimum 24 months follow-up, but with a substantially higher graft failure rate of about 23.9% [59]. Interestingly, the rupture of the graft is not always correlated with worse outcomes [54]. Consequently, SCR is believed to act as a mechanical spacer and thus improve shoulder kinematics [58,60]. This assumption, however, was disproved by a recent study that suggested that SCR may not depress the humeral head during functional abduction, and that the postoperative improvements in subjective and clinical outcomes may be affected by mechanisms other than changes in shoulder kinematics [61]. These findings are in line with a publication of Greiner et al. [62], who could not detect any differences in outcomes in the short term follow-up between the implantation of an SCR and partial infraspinatus repair in a matched-pair comparison of patients suffering from irreparable posterosuperior rotator cuff tears.

## 7. Subacromial Biodegradable Spacer

The SBS, also known as the balloon system, is a preshaped spacer made of a copolymer of poly-L-lactide, which biodegrades over approximately 12 months [63]. SBSs are arthroscopically inserted into the subacromial space and filled with saline solution, which subsequently depresses the humeral head. The biomechanical rationale behind performing this procedure in the setting of an irreparable RCT is to improve shoulder function by depressing the humeral head to a more central position on the glenoid, thus restoring the force couple and increasing deltoid load to improve the deltoid lever arm [11,63,64]. A recent biomechanical study confirmed these assumptions in a cadaveric model of an irreparable supraspinatus tear, showing that a balloon spacer restores intact-state glenohumeral contact pressures at most abduction angles, while also depressing the humeral head and increasing the deltoid load at time zero [11]. Moon et al. [65] summarized existing clinical results in a systematic review of seven outcome studies including 204 shoulders from 200 patients following subacromial spacer implantation due to irreparable posterosuperior RCT with Goutallier stage 3 and 4 fatty infiltration based on magnetic resonance imaging. Contraindications included cuff arthropathy stage Hamada 3 or higher, irreparable subscapularis tears, and pseudoparesis [66,67,68]. The mean age of patients was 68 years with a mean follow-up time of 19 months. All studies reported consistent and significant improvement in the total Constant Score or ASES Score over the duration of short-term follow-up with mean postoperative scores ranging between 60 and 70 points for the Constant Score and 70 and 80 points for the ASES Score. In a multi-center single-blinded, randomized controlled trial, Verma et al. reported comparable clinical outcomes between partial repairs, yet significantly greater forward elevation during early recovery as well as significantly shorter operating times [31]. However, another recent double-blinded randomized controlled trial comparing the arthroscopic debridement of the subacromial space with biceps tenotomy to an additional insertion of the subacromial balloon favored the group that received debridement and tenotomy only, as this group showed even slightly better outcomes after a 12-month follow-up [47]. Equally critical results were published by Garríguez-Pérez et al. at a comparable follow-up, reporting poor functional and satisfaction rates in a case series of 16 patients [9]. While a failure rate of only 3–13% had been reported at short-term follow-up [69], there are concerns regarding the effect beyond the biodegradation period of the spacer in the absence of mid- to long-term results in the literature to date [38]. This is reflected in single, low volume studies that report a preservation of a clinically relevant improvement in the postoperative outcome in only ~60% of the patients at a mid-term FU of 5 years [70]. In addition, an examination of reported adverse events such as foreign body reactions [71] and the risk of dislocations and more prospective data are needed to examine patient- and RCT-related factors predictive of the success of the procedure, providing more clarity regarding the perceived inconsistency in postoperative outcomes following the implantation of a balloon system in the contemporary literature (Table 6) [65,72,73].

## 8. Reverse Total Shoulder Arthroplasty

As surgeons have become more experienced with reverse total shoulder arthroplasty (RTSA), surgical indications have expanded over the past decade [74,75,76]. While irreparable RCTs with associated high-grade glenohumeral OA remain the most common indication for RTSA and show good to excellent outcomes [77], some authors suggest RTSA as a viable treatment option in patients with irreparable RCTs in the absence of glenohumeral OA due to its predictable pain relief and return to function (Table 7) [74,75,78]. Mulieri et al. [75] reported significant improvements in patient-reported outcomes (ASES, SST, VAS) in 60 shoulders following RTSA without significant OA after a minimum 2-year follow-up. The average patient age was 71 years and overall implant survivorship was 91% at a mean follow-up of 52 months [75]. Although RTSA in patients with low-grade OA leads to substantial subjective and functional improvement without clinical deterioration beyond 10 to 15 years, it is associated with a high complication rate of up to 39%, which ultimately compromises functional outcomes [69]. Special caution should be exercised when offering RTSA to young patients (especially those under the age of 65 years) as component loosening is the main cause for failure over time [79,80]. Additionally, patients without pseudoparesis and a good forward elevation preoperatively tend to have higher dissatisfaction rates because of the possible postoperative loss of active forward elevation compared to patients with pseudoparesis and a pre-existing limited range of motion [79].

## 9. Discussion

**Figure 1:**
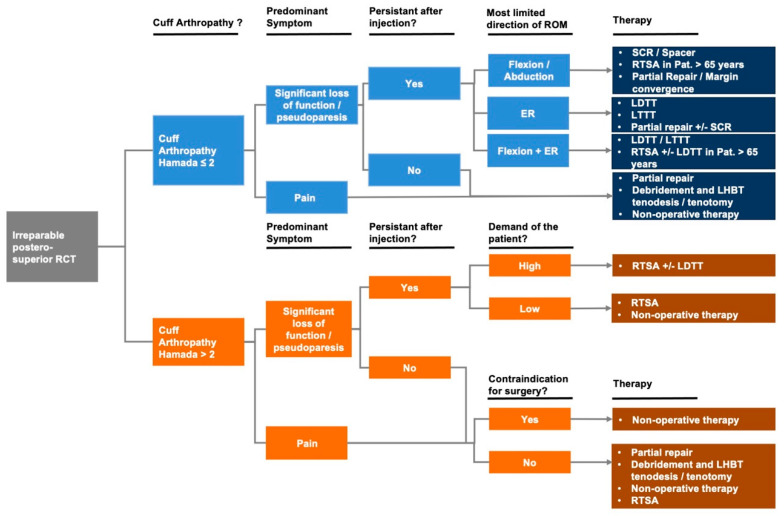
Treatment algorithm in the setting of irreparable posterosuperior rotator cuff tears. Abbreviations: ER, external rotation; SCR, superior capsule reconstruction; ROM, range of motion; RTSA, reverse total shoulder arthroplasty; LDTT, latissimus dorsi tendon transfer; LTTT, lower trapezius tendon transfer; LHBT, long head of the biceps tendon.

The optimal management of irreparable posterosuperior RCT remains a challenge for shoulder surgeons. If possible, the anatomic reconstruction of the torn rotator cuff should be attempted. As discussed above, if anatomic reconstruction is not feasible, several treatment modalities including non-operative and operative strategies are available and should be tailored to the patient’s individual preconditions and functional demand. Despite a large body of evidence on the management of irreparable posterosuperior RCT, there is still controversy about the ideal decision-making algorithm. In this current concepts review article, current evidence on surgical and non-surgical treatment strategies for irreparable posterosuperior RCT was summarized. As a result, a treatment algorithm with clinical steps and decision criteria is suggested to guide the shoulder surgeon in the decision-making for the management of irreparable posterosuperior RCTs (Figure 1).

In general, in the setting of an irreparable posterosuperior RCT, an initial non-operative treatment based on a standardized exercise program with the possibility for delayed, consecutive surgical treatment is recommended. However, purely non-operative treatment is often unsatisfactory and is mainly limited to patients not suitable for surgery or elderly patients with low functional demands. For active patients, a more selective approach is necessary as a posterosuperior RCT results in altered glenohumeral contact mechanics with the superior migration of the humeral head and accompanying force couple insufficiency. Consequently, abnormal glenohumeral contact pressure and microinstability cause rapid and early onset glenohumeral OA [7,81].

Thus, the evaluation of glenohumeral OA in patients with irreparable posterosuperior RCTs based on the classification proposed by Hamada et al. [82] should be the first step in the process of decision-making as multiple joint-preserving approaches such as tendon transfers, SCR, or spacer implantation are only suitable for patients with minimal osteoarthritic changes, typically defined as Hamada grade <2. The second step is to assess the main complaint of the patient. In patients reporting pain as their chief complaint, who only have low-grade glenohumeral OA without pseudoparesis, debridement followed by a partial rotator cuff repair to restore the force couple in combination with an LHBT tenotomy or tenodesis may be sufficient. The combination of these procedures reliably achieves pain relief and facilitates a high degree of functional compensation by a restoration of the force couple. However, as postoperative clinical outcomes may deteriorate over time, adequate patient counseling is crucial.

If a patient with low-grade glenohumeral OA presents with pseudoparesis or a significant loss of function as the chief complaint, the next diagnostic step is an injection of local anesthetics in patients, to determine whether the main reason for the functional deficit is primarily due to insufficient rotator cuff function or secondary to pain. In the case of a resolution of functional deficits upon injection, a partial repair combined with LBHT surgery as well as debridement are indicated. In the situation of persisting deficits following injection, it is important to determine the main limitation in the range of motion or main direction of pseudoparesis (i.e., flexion/abduction, external rotation, flexion + external rotation). In the case of deficits primarily in flexion or abduction, joint-preserving treatments such as a SCR and SBS implantation or (if possible) partial repair with margin convergence are recommended as possible treatment options, while an RTSA may also be considered in patients age > 65 years. However, although SCR and SBS implantation have become increasingly popular in recent years, as they preserve the native anatomy and do not compromise future treatment options, additional studies with long-term follow-up are required to sufficiently assess clinical outcomes, as existing early clinical outcome data are currently insufficient to provide final recommendations. In patients with deficits primarily in external rotation, an LDTT or LTTT is recommend as the treatment of choice, while a partial repair with a possible augmentation with an SCR may be a viable alternative. Of note, the clinical benefit of the combined LDTT and teres major tendon transfer (L’Episcopo technique) compared to the isolated LDTT is uncertain [44]. In patients with deficits in flexion that present with an additional positive external rotation lag sign, isolated tendon transfers as well as RTSA (in patients older than 65 years) +/− a combined LDTT are the recommended treatment options.

In patients with high-grade glenohumeral OA (i.e., Hamada > 2), only two surgical treatment options are recommended depending on the presence of pseudoparesis. A combined arthroscopic debridement, LHBT tenotomy/tenodesis, and partial rotator cuff repair is considered the first-line treatment in patients with good glenohumeral motion and no pseudoparesis, in case pain is the main symptom and/or functional deficits secondary to pain resolve upon the intra-articular injection of a local anesthetic. If severe functional deficits in any direction are present and persist after the injection of a local anesthetic in patients with high-grade glenohumeral OA, an RTSA is recommended with optional concomitant LDTT in patients with high physical demands and existing preoperative limitations of external rotation.

## 10. Conclusions

The management of irreparable posterosuperior RCTs remains a challenge for shoulder surgeons and treatment strategies should be tailored to the individual patient. In the non-functional, osteoarthritic shoulder, treatment strategies in the management of irreparable posterosuperior RCTs include debridement-based procedures and reverse total shoulder arthroplasty as the treatment of choice. Joint-preserving procedures aimed at restoring glenohumeral biomechanics and function should be reserved for the non-osteoarthritic shoulder. Prior to these procedures, however, patients should be counseled about deteriorating results over time. Recent innovations such as the SCR and the implantation of an SBS show promising clinical short-term results, yet current comparative studies indicate limited efficacy comparing the SCR or the in-space balloon to partial repair procedures. Taking the much higher costs for these innovative procedures into account, no fundamental recommendation can be given for either the SCR or the balloon. Future prospective comparative studies at long-term follow-up are required to delineate the sustainability of these procedures and derive stronger recommendations.

## Figures and Tables

**Table 1:** Advantages and disadvantages of non-operative treatment in the management of irreparable rotator cuff tears.

Advantages of Non-Operative Treatment	Disadvantages of Non-Operative Treatment
No risks related to surgery	Tear size progressing over time
	Discomfort and functional deficits may be retained
	Progression to cuff arthropathy

**Table 2:** Advantages and disadvantages of arthroscopic debridement, tenotomy/tenodesis of the long head of the biceps tendon and (reversed) subacromial decompression.

Advantages of Arthroscopic Debridement, Tenotomy/Tenodesis of the Long Head of the Biceps Tendon and (Reversed) Subacromial Decompression	Disadvantages of Arthroscopic Debridement, Tenotomy/Tenodesis of the Long Head of the Biceps Tendon and (Reversed) Subacromial Decompression
Short rehabilitation	High failure rate over time
Only minor surgical risk	Discomfort and functional deficits may be retained

**Table 3:** Advantages and disadvantages of partial rotator cuff repair.

Advantages of Partial Rotator Cuff Repair	Disadvantages of Partial Rotator Cuff Repair
Reliable short-term outcomes in patients without significant osteoarthritis	Clinical outcomes may deteriorate over time
	Long rehabilitation

**Table 4:** Advantages and disadvantages of tendon transfers.

Advantages of Tendon Transfers	Disadvantages of Tendon Transfers
Standardized surgical technique with long-term experiences with certain tendon transfers	High patient compliance necessary
Reasonable outcomes in a relatively young patient cohort	Long and complex rehabilitation
Restoration of active ROM	

**Table 5:** Advantages and disadvantages of superior capsular reconstruction (SCR).

Advantages of Superior Capsular Reconstruction (SCR)	Disadvantages of Superior Capsular Reconstruction (SCR)
Convincing short-term outcomes	Long-term outcomes not available
Less invasive surgery than tendon transfers	High percentage of graft retear rate
Preservation of anatomy	Limited cost-effectiveness compared to tendon transfers

**Table 6:** Advantages and disadvantages of the subacromial biodegradable spacer.

Advantages of the Subacromial Biodegradable Spacer	Disadvantages of the Subacromial Biodegradable Spacer
Only minor surgical risk	Long-term outcomes not available
Short rehabilitation	Biodegradation after 3 months
	Limited scientific evidence

**Table 7:** Advantages and disadvantages of reverse total shoulder arthroplasty.

Advantages of Reverse Total Shoulder Arthroplasty	Disadvantages of Reverse Total Shoulder Arthroplasty
Predictable and very good outcomes at short- and mid-term follow-up	Limited use in patients younger than 65 years
Relatively short rehabilitation	Functional deficits related to arthroplasty design
Considerable scientific evidence	Limited load-bearing capacity
